# A Pathogen Selection Tool for Surveillance of Acute Febrile Illness in Nigeria

**DOI:** 10.1093/cid/ciaf499

**Published:** 2025-11-20

**Authors:** Lauren P Courtney, Claire A Quiner, Cyril Erameh, Vivian Kwaghe, Jean H Kim, Jay Osi Samuels, Claire J Standley, Emmanuel A Oga

**Affiliations:** Solutions, RTI International, Durham, North Carolina, USA; Solutions, RTI International, Durham, North Carolina, USA; Institute of Viral and Emergent Pathogens Control and Research, Irrua Specialist Teaching Hospital, Irrua, Nigeria; Internal Medicine, University of Abuja Teaching Hospital, Gwagwalada, Federal Capital Territory, Nigeria; Solutions, RTI International, Durham, North Carolina, USA; Laboratory Services, APIN Public Health Initiative, Nigeria; Center for Global Health Science and Security, Georgetown University, Washington, District of Columbia, USA; Heidelberg Institute of Global Health, University of Heidelberg, Heidelberg, Germany; Solutions, RTI International, Durham, North Carolina, USA; ClineEpi Partners, Columbia, Maryland, USA

**Keywords:** acute febrile illness (AFI), infectious disease surveillance, TaqMan array card, pathogen selection

## Abstract

**Background:**

Acute febrile illness (AFI) is a common manifestation of infectious diseases and a frequent reason for seeking medical care. Because AFI is a likely clinical presentation of several infectious diseases of public health significance, inadequate diagnostic capacity can delay outbreak detection and response. The Surveillance of Acute Febrile Illness Aetiologies in Nigeria (SAFIAN) study aimed to investigate the infectious causes of AFI in Nigeria.

**Methods:**

We used a TaqMan Array Card (TAC) technology, one of several commercially available tools based on reverse-transcription polymerase chain reaction, which can be customized for simultaneous detection of multiple pathogens. Researchers must prioritize pathogens to target for surveillance, given that it is not feasible or cost-effective to test all pathogens endemic in a region.

**Results:**

We developed a 6-step model for pathogen selection for AFI surveillance studies. These 6 steps included (1) defining the study objectives; (2) identifying a global list of potential pathogens compatible with study objectives; (3) evaluating transmission potential of selected pathogens in region(s) of interest; (4) defining the pathogen inclusion and exclusion criteria based on surveillance interests; (5) applying inclusion and exclusion criteria to rank pathogen importance based on evidence of endemicity or alignment with surveillance interest; and (6) selecting the final list of pathogens for surveillance. Using this model, we identified and evaluated 69 potential pathogens and customized a TAC panel with 25 targets.

**Conclusions:**

Despite the lack of external feedback, we posit that this model for pathogen selection can be applied to future AFI surveillance studies in resource-constrained settings.

This article presents a 6-step, data-driven framework for optimizing multi-pathogen screening tools, aligning pathogen selection with transmission potential. The adaptable, user-friendly tool enhances surveillance in low-resource settings, supporting early outbreak detection and response before widespread transmission occurs.

## Introduction

Early detection and identification of infectious diseases of epidemic potential is critical for public health response and for preventing future epidemics. Although surveillance and routine screening is the predominant approach facilitating early identification, public health professionals and researchers in low-resource settings need to select a manageable list of pathogens for active routine surveillance because of resource constraints [1].

Acute febrile illness (AFI) is a common manifestation of infectious ailments. The challenge posed by undifferentiated AFI is that it can sometimes be the first indication of infection by an emerging or epidemic-prone pathogen that may not be included in routine testing. Additionally, in malaria-endemic settings, like Nigeria, AFI cases are often presumptively diagnosed as malaria [2–5]. Despite global advances in laboratory diagnostics, many patients with undifferentiated AFI continue to go undiagnosed or be misdiagnosed. Even with multi-pathogen detection, AFI research often detects only half of participants [6]. Given limited resources, laboratories and surveillance programs must decide which pathogens to include in regular screening. Public health surveillance must balance limited resources between testing for common endemic diseases and rarer emerging threats with high-impact potential. The 2014–2016 Ebola outbreak in West Africa illustrates the risk of delayed outbreak detection, as 4 months passed before Ebola virus was identified—partly because of its exclusion from routine screening amid limited testing resources [7, 8].

Multi-pathogen screening and diagnostic tools such as the TaqMan Array Card (TAC) are helpful for simultaneous surveillance of multiple priority pathogens in resource-constrained settings, but they should be optimized for the appropriate array of pathogens. This way, these tools can be important sources of information for public health preparedness efforts. The existence of several commercial products that allow for broad screening of multiple pathogens within a single assay raises the possibility of conducting multi-pathogen surveillance for a large number of targets using a single, commercially available resource; some of the products also allow for customization of the assay targets.

The AFI literature includes a wide spectrum of pathogens; Rhee et al identified 105 pathogens included for surveillance in AFI research in a scoping review of 190 publications from 2005 to 2017 [9]. Despite the wide diversity of potential pathogens, AFI studies often provided limited to no details on how their surveillance targets were selected [10, 11]. Some studies prioritized pathogens that have been previously detected in a region [12, 13]; others focused on syndromic surveillance (eg, diarrheal disease or AFI surveillance) [10].

Although most published AFI studies have not published their pathogen selection process, other disease prioritization efforts offer valuable insights. In response to the need for standardized prioritization, the World Health Organization (WHO) convened experts in 2015 to recommend best practices, including 2 rounds of the Delphi technique process followed by a rigorous criterion-based assessment [14]. The European Centre for Disease Prevention and Control (ECDC) reviewed 17 prioritization studies to inform the development of its own decision-making tool for emergency preparedness [15]. In parallel, the US Centers for Disease Control and Prevention (CDC) created the One Health Zoonotic Disease Prioritization tool, which has been implemented in countries such as Uganda [16], Somalia [17], and India [18]. Another example is the One Health systems assessment for priority zoonoses, which incorporates a consensus-driven prioritization step involving multiple government ministries [19].

Some public health researchers have applied and published formal disease prioritization methods to guide decision-making. Documented approaches include multicriteria decision analysis [20–23], Delphi techniques [1], bibliometric indexes [24], and structured questionnaires [23]. These methods frequently rely on expert input, such as interviews and scoring frameworks, and are often resource intensive. Although these approaches are effective, they vary in data sources, prioritization criteria, and intended outcomes, and they often require substantial time, expertise, and funding to implement.

This article presents the 6-step process used by the Surveillance of Acute Febrile Illness Aetiologies in Nigeria (SAFIAN) study to optimize the multi-pathogen screening tool by developing a systematic approach for pathogen selection. Using publicly available data, this method is a multicriteria analysis that matches pathogen selection with transmission potential of pathogens, designed to be simple for researchers and adaptable for low-resource settings.

## METHODS

**Figure 1.**
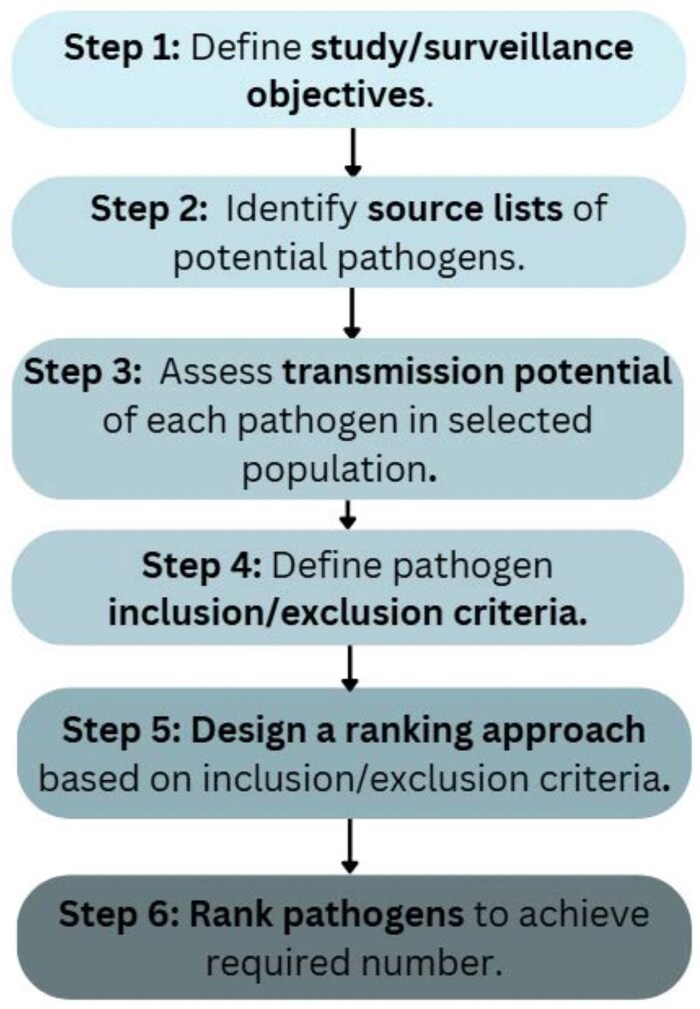
Approach to the pathogen selection process for researchers.

We followed a systematic process to assess various pathogens rapidly, using only publicly available data to select 25 of the highest-priority pathogens for inclusion on a TAC testing panel for surveillance of AFI in Nigeria. Building upon the infectious disease “risk-ranking” process used by ECDC to develop a list of priority pathogens, SAFIAN's approach used a scoring system with similar criteria. As shown in Figure 1, we used a 6-step model that simplified this process for easier adoption.

### Step 1: Define Study/Surveillance Objectives

Specific and well-defined objectives are necessary to guide researchers’ decision-making about data collection plans and pathogen selection [25]. We defined our surveillance objectives as follows:

The primary objective was to identify pathogens of high epidemiologic consequence, high morbidity, and high mortality that have potential transmission to humans in Nigeria, including those for which no prior surveillance efforts exist.

Secondary objectives were (1) to generate comparable data to other AFI surveillance studies in the region by including some pathogens from such studies for comparability; and (2) to represent diverse pathogen categories of clinical and epidemiological significance (eg, viral, protozoan, bacterial).

### Step 2: Identify Source Lists for Pathogens

Various pathogen source lists were available in the literature, which we reviewed for this step. We used source lists to compile a master list of pathogens consistent with the study objectives. Beginning with the WHO Integrated Disease Surveillance and Response (IDSR) priority pathogen list [26] for Nigeria, we included the “epidemic-prone” pathogens (n = 13). We added the National Institute of Allergy and Infectious Diseases’ (NIAID) category A and B pathogens (n = 54) [27], which, while developed in a US context, reflect pathogens of high epidemiologic consequence and significant morbidity and mortality—criteria relevant to AFI surveillance in Africa. Finally, we included pathogens included in other AFI studies to allow data comparison (n = 31). With overlap from these 3 sources, we included 69 pathogens.

### Step 3: Assess Transmission Potential in Geographic Area of Interest

We assessed pathogens on the master list to determine transmission pathways, previous accounts of detection in the study region, and ecological considerations of host/vector (where applicable). This step was conducted via a literature review, with outcomes defined by each of the following levels of detection for each pathogen: (1) capability for human-to-human transmission; (2) previous detection in humans in Nigeria; (3) previous detection of the pathogen in nonhuman hosts in Nigeria, such as rodents or ticks; (4) previous detection of the pathogen's vector or reservoir in Nigeria; and (5) ecological suitability for its vector or reservoir in Nigeria.

Pathogens that did not demonstrate previous human-to-human transmission were included only if the environment was suitable for the disease vector.

### Step 4: Define Inclusion Criteria

Inclusion criteria were established to select and rank pathogens. This process used multicriteria decision analysis, which involved setting each criteria according to the study's goals. These criteria were crafted to evaluate how well each pathogen aligned with the study's objectives.

Once criteria were defined, publicly available data sources were identified to ensure that each factor could be accurately assessed. Four criteria were defined for the SAFIAN study (Table 1) based on the study objectives from step 1.

**Table 1.** SAFIAN Pathogen Inclusion Criteria

Criterion Number	Inclusion Criteria	Study Objectives Assessed	Definition	Data Source
Criterion 1	High epidemiologic consequence	1	Pathogen has a high outbreak potential per the IDSR or NIAID definitions.	Nigeria IDSR “epidemic-prone” pathogens [26, 28]; NIAID category A pathogens [27]
Criterion 2	High morbidity and mortality	1	Pathogen has a high likelihood of potential for morbidity or mortality per the NIAID definition.	NIAID category A and B pathogens [27]
Criterion 3	Potential for transmission in Nigeria	1	Pathogen has potential for transmission based on route of transmission, previous detection, presence of its vector/reservoir, or ecological suitability for its host/reservoir but have scarcely or never been identified in previously studies or public case reports.	Assessment performed in step 3; literature review [29]
Criterion 4	Priority diseases from other regional studies	2	Pathogens included in at least one other AFI study in the country or region.	NCDC surveillance research protocol^a^; meta-analysis of AFI [6, 12]

Abbreviations: AFI, acute febrile illness; IDSR, World Health Organization Integrated Disease Surveillance and Response; NCDC, Nigerian Centers for Disease Control; NIAID, National Institute of Allergy and Infectious Diseases; SAFIAN, Surveillance of Acute Febrile Illness Aetiologies in Nigeria.

^a^Unpublished (2022).

Criterion 1 identified pathogens with high epidemiologic consequence, based on published sources, including Nigeria's IDSR epidemic-prone pathogens and the NIAID category A pathogen list. Category A pathogens are defined by NIAID as organisms/biological agents that pose the highest risk to national security and public health because they can be easily disseminated or transmitted from person to person, result in high mortality rates, have the potential for major public health impact, might cause public panic and social disruption, and require special action for public health preparedness.

For criterion 2, NIAID's category A and B pathogens were used to indicate pathogens with high morbidity and mortality. Category B pathogens were included but given a lower priority because they include pathogens with only moderate morbidity and mortality.

Criterion 3 evaluated each pathogen's potential for transmission in Nigeria. This was based on the pathogen's route of transmission, previous detection (molecular or serological) in humans (or nonhumans in area of interest), presence of its vector/reservoir in relevant geographic areas, and the ecological suitability for host/reservoir of a pathogen [29]. As noted previously, this assessment was based on a literature review, published separately [29].

Criterion 4 evaluated whether the pathogen was included in other AFI studies in the region to support comparability of study results with similar regional studies. This was determined through a review of several recent studies conducted in the region. Criterion 4 was linked to subobjective 2.

### Step 5: Design Ranking Approach Based on Inclusion and Exclusion Criteria

We developed a prioritization process for each criterion outlined in step 4 to rapidly assess the relative importance of each pathogen to the study objectives. Each pathogen was evaluated against 4 criteria. Pathogens that did not meet at least one inclusion criterion were excluded. The remaining pathogens were prioritized according to the number of criteria met. While meeting criteria 3 was mandatory for inclusion, pathogens that also met criteria 1 or 2 were given priority due to their direct relevance to the study's objectives. Criterion 4 was linked to secondary objectives.

For criteria 1 and 2, we employed NIAID's established definitions and inclusion standards as a foundation for the assessment, ensuring that our analysis was grounded in recognized credibility for pathogens with significant epidemiological impact and high morbidity and mortality rates. Pathogens classified under NIAID category A were considered especially relevant.

Criterion 3 served as a pivotal filter in our selection process. It was the first step in eliminating pathogens lacking transmission potential within Nigeria. Inclusion was based on documented evidence of detection in the country and the ecological context of the host or vector.

Criterion 4 focused on comparability across studies and was informed by literature reviews and consultation with other regional researchers. Pathogens were prioritized if they had been included in other AFI research in Nigeria or neighboring countries.

### Step 6: Rank Pathogens to Achieve Required Number

After evaluating all criteria, the research team conducted a final assessment based on the pathogens’ ranked order, determined by the application of criteria in step 5. This step involved critical decision-making to refine the prioritization of pathogens, considering study limitations, such as the number of wells on a TAC and budgetary constraints.

In addition to these factors, the team accounted for these considerations:

- Technology: The capacity of the TAC and the multiplexing platform used, which could influence the development of multiplex reverse-transcription polymerase chain reaction (PCR) assays, including capacity of the TAC platform to include the pathogens.
- Sensitivity versus specificity: Balancing the trade-offs between sensitivity and specificity. When developing the TAC layout, the team allocated double spots per pathogen to increase detection chances at the expense of reducing the number of pathogens that could be included.
- Distinct symptomology: Pathogens with highly specific symptoms were excluded, as they are unlikely to present as undifferentiated fever.
- Strain variation: The genetic diversity of target pathogens, necessitating the inclusion of multiple strains for comprehensive detection.
- Controls: The availability of positive and negative controls for assay development and testing, particularly when creating new primer sets or employing serological methods.
- Biospecimen feasibility: Researchers determined that accurate detection tools were available for the selected pathogens and specimen types. For example, respiratory pathogens (eg, severe acute respiratory syndrome coronavirus 2 [SARS-CoV-2]) may require a nasal swab for more sensitive detection, whereas Orthopox viruses are most easily detected in a swab of a pox lesion. The team had to determine if they would be able to collect the appropriate biospecimen sample.
- Diversity of representation of pathogens: This was linked to study subobjective 2, which sought to ensure a diverse representation of viral, protozoan, and bacterial pathogens.

As we reviewed each pathogen's ranking, we conducted a detailed stepwise review of each criterion and these additional considerations. In consultation with local clinical stakeholders, our team's subject-matter experts made informed decisions to prioritize the final selection, ensuring a robust and representative pathogen panel for our study. These additional considerations factored into the final decision-making in enabling to team to reduce the list of pathogens.

## RESULTS

**Figure 2.**
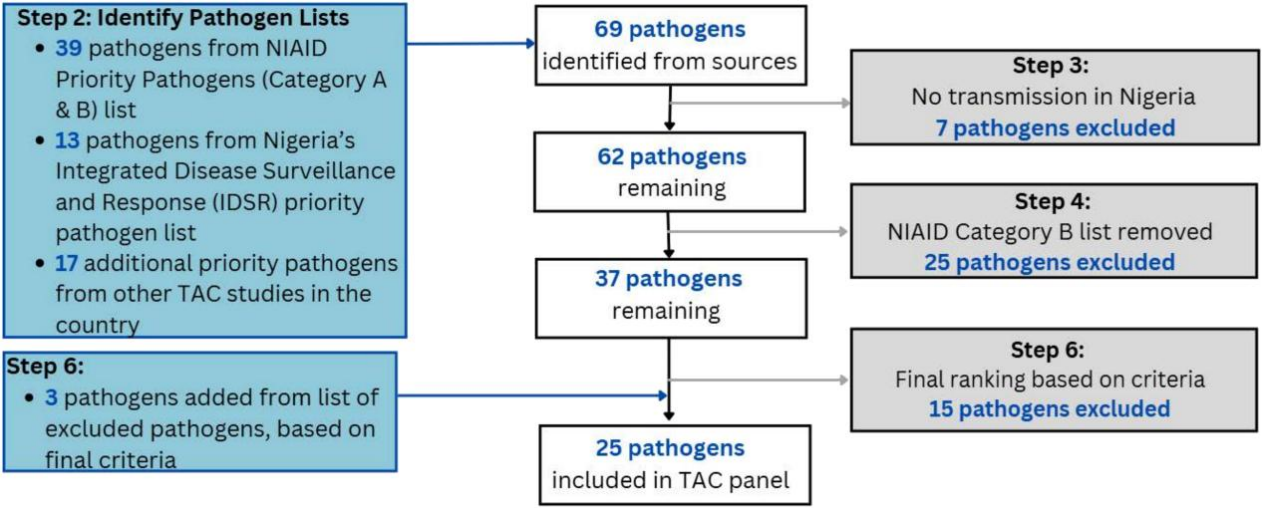
Flowchart documenting the approach to selecting pathogens for Surveillance of Acute Febrile Illness Aetiologies in Nigeria (SAFIAN). Abbreviations: NIAID, National Institute of Allergy and Infectious Diseases; TAC, TaqMan Array Card.

Beginning with 69 pathogens from 3 data sources, we were limited to 25 molecular targets for inclusion on TAC. This total number included duplicated targets to ensure additional screening sensitivity and overall improved quality. Results from our application of inclusion and exclusion criteria are shown in Figure 2, which reached the final list as shown in Table 2, and excluded pathogens in Table 3.

**Table 2.** SAFIAN-Selected Pathogens Using the 4 Selection Criteria and Pathogen Type

Surveillance Target (Pathogen)	Criterion 1	Criterion 2	Criterion 3	Criterion 4	Pathogen Type
Dengue fever (dengue virus)	√	√	√	√	Viral
Lassa fever (Lassa virus)	√	√	√	√	Viral
Mpox (Monkeypox virus)	√	√	√	√	Viral
Pan-filovirus (Ebola virus, Marburg virus)	√	√	√	√	Viral
Rift Valley fever	√	√	√	√	Viral
Chikungunya (chikungunya virus)	√	√	√	√	Viral
Yellow fever (yellow fever virus)	√	√	√	√	Viral
Zika fever (Zika virus)	√	√	√	√	Viral
Crimean-Congo hemorrhagic fever	√	√	√	×	Viral
Meningitis (*Neisseria meningitidi*s)	√	√	√	√	Bacterial
Plague (*Yersinia pestis*)	√	√	√	√	Bacterial
Pan-*Salmonella* (*Salmonella* spp.)	√	√	√	√	Bacterial
Pan-Orthopox virus (VACV-like viruses, Parapox, Cowpox)	√	√	√	×	Viral
Hantavirus disease (hantaviruses)	√	√	√	×	Viral
Brucellosis (*Brucella* spp.)	×	√	√	√	Bacterial
Q fever (*Coxiella burnetii*)	×	√	√	√	Bacterial
West Nile (West Nile virus)	√	√	√	×	Viral
Hepatitis E (hepatitis E virus)	×	×	√	√	Viral
O’nyong’nyong fever (O’nyong’nyong virus)	×	×	√	√	Viral
Leishmaniasis (*Leishmania* spp.)	×	×	√	√	Protozoan
Malaria (*Plasmodium* spp.)	×	×	√	√	Protozoan
Trypanosomiasis (*Trypanosoma brucei*)	×	×	√	√	Protozoan
Bartonellosis (*Bartonella* spp.)	×	×	√	√	Bacterial
Leptospirosis (*Leptospira* spp.)	×	×	√	√	Bacterial
Rickettsial infections (*Rickettsia* spp.)	×	×	√	√	Bacterial

**√** indicates that the criterion was met; × indicates that the criterion was not met.

Abbreviations: SAFIAN, Surveillance of Acute Febrile Illness Aetiologies in Nigeria; VACV, Vaccinia Virus.

**Table 3.** Additional Pathogens Reviewed and Not Selected Using the 4 Selection Criteria and Pathogen Type

Surveillance Target (Pathogen)	Criterion 1	Criterion 2	Criterion 3	Criterion 4	Pathogen Type
Caliciviruses	×	×	√	×	Viral
California encephalitis	×	×	NA	×	Viral
Chapare virus	√	√	NA	×	Viral
Eastern equine encephalitis	×	×	×	×	Viral
Guanarito virus	√	√	×	×	Viral
Hepatitis A	×	×	√	√	Viral
Japanese encephalitis virus	×	×	√	×	Viral
Junín virus	√	√	×	×	Viral
La Crosse encephalitis	×	×	√ (1)	×	Viral
Lujo virus	√	√	NA	×	Viral
Machupo	√	√	×	×	Viral
Nipah	×	×	×	√	Viral
Poliovirus	√	√	√	×	Viral
Rabies (rabies virus)	√	×	√	×	Viral
Rubeola (measles virus)	√	×	√	×	Viral
SARS-CoV-2	√	×	√	√	Viral
St. Louis encephalitis virus	×	√	√ (1)	×	Viral
Venezuelan equine encephalitis	×	×	×	×	Viral
Western equine encephalitis	×	×	NA	×	Viral
*Balamuthia mandrillaris*	×	√	×	×	Protozoan
*Cryptosporidium parvum*	×	×	√	×	Protozoan
*Cyclospora cayatanensis*	×	×	√	×	Protozoan
*Entamoeba histolytica*	×	×	√	×	Protozoan
*Giardia lamblia*	×	×	√	×	Protozoan
*Naegleria fowleri*	×	×	√	×	Protozoan
*Toxoplasma gondii*	×	×	√	×	Protozoan
Microsporidia	×	×	√	×	Fungal
*Bacillus anthracis* (anthrax)	√	×	√ (2)	×	Bacterial
*Burkholderia mallei* (glanders)	×	√	√ (1)	√	Bacterial
*Burkholderia pseudomallei* (melioidosis)	×	×	√	×	Bacterial
*Campylobacter jejuni*	×	×	√	×	Bacterial
*Chlamydia psittaci* (psittacosis)	×	×	√ (1)	×	Bacterial
Cholera	√	×	√	×	Bacterial
*Clostridium botulinum* toxin (botulism)	√	×	√	×	Bacterial
Diarrheagenic *Escherichia coli*	×	×	√	×	Bacterial
*Francisella tularensis* (tularemia)	√	√	√ (1)	×	Bacterial
*Listeria monocytogenes*	×	×	√	×	Bacterial
*Orientia tsutsugamushi*	×	×	√ (2)	√	Bacterial
Pathogenic *Vibrio* spp.	×	×	NA	×	Bacterial
*Shigella* spp.	√	×	√	×	Bacterial
*Streptococcus pneumoniae*	√	×	√	√	Bacterial
Typhoid fever (*Salmonella* serotypes Typhi and Paratyphi)	×	×	NA	√	Bacterial
*Yersinia enterocolitica*	×	×	√	×	Bacterial

**√** indicates that the criterion was met; × indicates that the criterion was not met. 1: Pathogen not detected in humans, animals, or vector in Nigeria, but pathogen has potential since vector is present. 2: Pathogen not detected in humans or animals in Nigeria, vector not detected in Nigeria, but ecological conditions are suitable for the vector [29].

Abbreviations: NA, review not available; SARS-CoV-2, severe acute respiratory syndrome coronavirus 2.

For criteria 1 and 2, pathogens of high epidemiologic consequences and high morbidity and mortality were prioritized. Pathogens on NIAID's category B list described pathogens with moderate epidemiologic consequences and morbidity. Thus, in step 4, we excluded 25 category B pathogens that were not retained based on other criteria to reduce the list.

For criterion 3, the initial exclusion process in this decision-making tool resulted in 19 pathogens removed because they have no likelihood of transmission in Nigeria. For example, several arenaviruses (Chapare, Guanarito, Junín, and Eastern equine encephalitis virus), which can cause viral hemorrhagic fever, were excluded because they have only been found on other continents, and hosts (eg, *Calomys* rodents) are not present in the country, leaving no potential for transmission in Nigeria, through our One Health Transmission Potential framework [29].

Based on criterion 4, some pathogens from the category B list, which may otherwise have been excluded for lower mortality and morbidity and not considered of “high epidemiological consequence,” were prioritized for inclusion because these pathogens were determined to be of epidemiologic research interest based on the literature. For example, West Nile virus was shown to be understudied but with significant evidence of presence in Nigeria; *Rickettsia* was included due to interesting results from other AFI studies [10, 13].

Additional pathogens were excluded due to distinct symptomology. For example, measles was excluded because there is significant clinical and public knowledge of illness (including routine vaccination), existing surveillance, and it is primarily diagnosed in pediatric age groups. Because of this heightened consciousness regarding measles, it is not often misdiagnosed, so we prioritized other pathogens for inclusion on the TAC. Pathogens from the IDSR priority pathogen list that were marked for eradication only but not as epidemic prone were excluded; examples include diphtheria, pertussis, lymphatic filariasis, neonatal tetanus, and leprosy. We excluded SARS-CoV-2 because our data collection period began after the pandemic's peak, and we anticipated a smaller number of cases. To maximize use of study resources, we did not include a respiratory panel and only collected samples via blood or pox lesions, excluding nasal swabs.

The TAC includes 48 wells per sample. Two wells were used for the 18S extraction/PCR control. The remaining 46 wells could be used for pathogen detection. To ensure sensitivity and for quality control, we initially planned to include duplicate wells for each pathogen for a total of 23 pathogens. However, to allow for more pathogens, we allowed duplicate wells for 21 pathogens and single wells for 4 pathogens, thus providing 25 pathogens. Singlets included pan-Orthopox virus (including monkeypox virus and cowpox virus), monkeypox virus, *Neisseria meningitidis*, and *Plasmodium* spp. Monkeypox virus was a singlet because it was also present in pan-Orthopox. *Neisseria meningitidis* was a singlet because of distinct symptoms and being lower priority. *Plasmodium* spp. was a singlet because it is commonly tested by hospitals.

## DISCUSSION

This pathogen prioritization tool, characterized by rigorous selection criteria, enabled evidence-based evaluation of 69 pathogens and the development of a customized 25-target TAC panel for the SAFIAN study. Employing criterion-based methodology with publicly available published literature as the primary source facilitated efficient decision-making processes. While this process was applied to develop a TAC-based molecular panel, the same framework could be adapted for serological studies to prioritize pathogens for exposure assessment. Using publicly available data and published results, the use of this tool ensured resource efficiency and supported scientifically grounded data, which could allow for replicable decision-making across diverse settings. For example, step 1 began by developing the list of potential pathogens with 2 key data sources. These lists were previously vetted by WHO [26] and NIAID [27]. Adopting lists gathered from other experts offers efficiencies for AFI researchers.

Pathogen selection methods published by organizations such as WHO and other researchers are often resource-intensive and typically suited for large-scale evaluations, and they rely on mixed methodologies though they yield comprehensive and well-synthesized results. In contrast, the SAFIAN decision-making tool was developed with practical constraints in mind, opting for publicly available online data in place of expert interviews to conserve time and resources. This approach enhances cost-effectiveness and accessibility, particularly in resource-limited settings, by avoiding labor-intensive methods used in other AFI studies. The SAFIAN tool offers a flexible, criterion-based approach that supports algorithmic decision-making and is particularly useful in resource-limited settings. Although similar to the ECDC's risk-ranking framework [15], SAFIAN includes an additional step to assess transmission potential within the geographic area of interest. Its design eliminates the need for a broad panel of experts, making it accessible to researchers with limited funding and well-suited for early-stage protocol development. By leveraging scientifically sourced data, the tool ensures a credible and balanced approach to pathogen selection.

The use of TAC technology for simultaneous pathogen detection is a key strength of this approach, offering high-throughput molecular diagnostics suited to AFI surveillance. However, researchers should carefully consider whether TAC is the most appropriate tool for each pathogen in a surveillance study, especially when clinically validated and readily available diagnostic methods exist. For example, malaria is often accurately diagnosed through rapid diagnostic tests or microscopy, which are widely used in clinical settings and offer reliable detection of acute infection. Pathogen-agnostic methods, such as metagenomic sequencing, also offer a powerful approach, and may be particularly appropriate for contexts where the detection of novel or highly genetically diverse pathogens is a priority—especially those that may evade identification through a targeted approach. However, selection of the diagnostic platform should also consider feasibility and sustainability in resource-limited environments. To address these considerations, pathogen inclusion was guided by biospecimen feasibility, ensuring that appropriate sample types (eg, blood or pox swabs) could be collected for optimal detection. Where molecular detection was less sensitive or less practical, alternative diagnostic strategies were considered or flagged for future evaluation.

### Limitations

One limitation is the lack of external feedback. Validation of the tool through external subject-matter expert review was not feasible within the scope of this project. Additionally, there was limited literature on the methodological guidance for pathogen selection from other AFI surveillance studies to support our process, specifically concerning which criteria were used for pathogen selection. Last, the tool's reliance on existing pathogen lists may introduce bias, as these sources may not fully reflect local epidemiological patterns.

## CONCLUSIONS

Given the increasing concern surrounding numerous pathogens and the widespread adoption of multiplex testing platforms like TAC technologies, there is a growing need for an efficient, easily implementable, and robust decision-making tool to guide pathogen selection. The methodology outlined in this study facilitated a systematic ranking of pathogens based on 6 steps, with selection criteria and data from diverse sources. By integrating scientific literature and other published resources, we effectively prioritized pathogens for inclusion via a rapid assessment process. This adaptable decision-making tool empowers researchers to ensure evidence-based pathogen selection by applying rigorous and thoughtful criteria aligned with the study objectives. The proposed 6-step model for pathogen selection holds promise for researchers operating under resource constraints, offering a valuable tool for designing customizable screening efforts for AFI surveillance studies.
